# Outcomes with intracytoplasmic sperm injection using fresh vs. frozen elongated spermatids from microsurgical testicular sperm extraction

**DOI:** 10.1016/j.xfre.2026.08.005

**Published:** 2026-08-25

**Authors:** Jason E. Elliott, Christopher Labos, Jim S. Meriano, Keith Jarvi, Ellen M. Greenblatt

**Affiliations:** a Mount Sinai Fertility (MSF), Sinai Health System, Toronto, Ontario, Canada; b Department of Obstetrics and Gynaecology, Temerty Faculty of Medicine, University of Toronto, Toronto, Ontario, Canada; c Department of Obstetrics, Gynecology and Reproductive Sciences, Max Rady College of Medicine, Rady Faculty of Health Sciences, University of Manitoba, Winnipeg, Manitoba, Canada; d Department of Global and Public Health, McGill University, Montreal, Quebec, Canada; e TRIO Fertility, Toronto, Ontario, Canada; f Department of Surgery – Division of Urology, Sinai Health System, Toronto, Ontario, Canada; g Division of Urology, Department of Surgery, Temerty Faculty of Medicine, University of Toronto, Toronto, Ontario, Canada

**Keywords:** Spermatids, intracytoplasmic sperm injection, microsurgical testicular sperm extraction, live birth rates

## Abstract

**Objective:**

To evaluate the reproductive outcomes of intracytoplasmic sperm injection (ICSI) using fresh and frozen-thawed elongated spermatids obtained from microsurgical testicular sperm extraction (mTESE) from men with nonobstructive azoospermia.

**Design:**

Retrospective cohort study.

**Subjects:**

A total of 256 infertile men underwent one or more mTESE procedures between 2004 and 2017, with the intention of attempting to conceive via ICSI with their female partners.

**Exposure:**

Intracytoplasmic sperm injection was performed using fresh or frozen-thawed elongated spermatids.

**Main Outcome Measures:**

Sperm retrieval rate, fertilization rate, blastulation rate, embryo transfer rate, positive β-human chorionic gonadotropin level, clinical pregnancy rate, live birth rate, miscarriage rate.

**Results:**

A total of 256 men with nonobstructive azoospermia underwent 292 mTESE procedures. Of these, 90 men underwent 99 mTESE procedures in which only elongated spermatids were retrieved, and ICSI was performed later. One hundred-thirty eight ICSI cycles were performed using fresh or frozen elongated spermatids. Overall, fertilization and blastulation rates were 35.3% and 19.3%, respectively. Clinical pregnancy and live birth rates were 4.3% and 2.9%, respectively. There were no differences between live birth outcomes with fresh and frozen elongated spermatids. This relationship persisted after adjusting for male age at the time of mTESE, female age at the time of ICSI, and number of oocytes inseminated (fresh vs. frozen-thawed, odds ratio = 0.40 [0.03–5.33]).

**Conclusion:**

A nonsignificant difference in reproductive outcomes between fresh and frozen-thawed elongated spermatids can facilitate scheduling of the mTESE procedure before or concurrently with ovarian stimulation cycles without compromising patient outcomes. However, the overall very poor reproductive outcomes with elongated spermatids suggest that very thorough counseling of patients utilizing spermatids should be mandatory, and use of donor sperm should be strongly considered to improve chances of a live birth.

Male factors affect up to 50%–60% of couples with infertility; of these, 9%–15% will have azoospermia, which can be obstructive (OA) or nonobstructive (NOA) (1, 2). Before the development of intracytoplasmic sperm injection (ICSI), men with NOA had few options other than the use of donor sperm for conception (3, 4). With ICSI, surgically obtained sperm can be used for in vitro fertilization (IVF). When using microsurgical testicular sperm extraction (mTESE), the current standard approach for men with NOA, both mature and immature sperm (spermatids) can be retrieved (5). Spermatids can be characterized as either elongated or round, differentiating between more and less mature stages, respectively (6, 7, 8). Published reports show fertilization rates and subsequent fertility success with mature testicular sperm are superior to those utilizing spermatids in men with NOA (9). Accordingly, mature sperm is preferred; however, there are situations where a couple may consider use of spermatids, such as when use of donor sperm is unacceptable, and spermatids remain the only possible way to achieve biological children. Although there is evidence to suggest no difference in success with fresh mature sperm vs. frozen-thawed mature sperm (10, 11, 12, 13, 14, 15, 16, 17, 18, 19, 20, 21, 22), some reproductive urologists prefer fresh sperm to be used for ICSI (23). However, despite this preference, use of frozen-thawed sperm in ICSI from a prior mTESE procedure occurs more than 60% of the time (23). In this context, it would be helpful to understand if the outcome of ICSI using elongated spermatids is influenced by their fresh or frozen-thawed status.

To our knowledge, there is only one very small study directly assessing ICSI outcomes using fresh vs. frozen-thawed spermatids after mTESE, with no difference reported for elongated spermatids (24). All other reported studies either used exclusively fresh spermatids (25, 26, 27, 28, 29, 30, 31, 32, 33, 34, 35, 36, 37, 38, 39, 40, 41, 42, 43, 44, 45, 46) or frozen spermatids (47, 48, 49, 50, 51, 52). This study aimed to compare contemporary reproductive outcomes associated with ICSI cycles utilizing fresh and frozen-thawed elongated spermatids obtained via mTESE in men with NOA. We hypothesized that like mature sperm, elongated spermatids would be similarly unaffected by cryopreservation status and reproductive outcomes would be similar. These results will help guide clinical practice for clinics using spermatids for reproductive use.

## Materials and methods

### Study design/patient selection

This is a retrospective cohort study focusing on men who underwent mTESE procedures performed for NOA through Mount Sinai Hospital (MSH), Toronto, Canada, between January 2004 and December 2017, in whom only elongated spermatids were retrieved and subsequently used in ICSI cycles performed at two fertility clinics in Toronto (Mount Sinai Fertility and TRIO Fertility). Patient-specific data obtained from the MSH patient chart records included preoperative hormonal testing, diagnostic imaging, testicular histology from prior biopsies (if performed) and genetic assessments, as well as surgical procedure and intraoperative mTESE sperm retrieval details.

Ethical approval for this study was obtained from the Research Ethics Board at Mount Sinai Hospital (14-0342-E and 17-0098-C).

### Sperm retrieval

Surgical sperm retrieval was performed over the study period by three subspecialty-trained urologists experienced in mTESE procedures. Testicular sperm/spermatids were obtained via mTESE performed under general anesthesia, as described previously (53). After examination by the surgeon under light microscopy in the operating room immediately after retrieval, the samples were placed in 1-mL vials containing modified human tubal fluid (mHTF) (Irvine Scientific, Irvine, CA) and transported immediately to the referring fertility clinic. In the two embryology laboratories, very similar processes of sperm preparation were undertaken, as follows: surgical mTESE samples were prepared by an embryologist upon arrival, with tissue placed into 1 mL of mHTF/10% LGPS (Life Global Protein Solution) (v/v) (Life Global Inc, Guilford, CT) and dissected using a 27 G ½ mL needle. Tissue was dissected extensively until microtubular contents were expelled into the media. Higher power microscopy with phase-contrast optics allowed the visualization of mature and immature sperm, which were also assessed under an inverted microscope (×400 magnification, Hoffman modulation optics). The prepared samples were then covered and left at room temperature until needed for fresh spermatid injection the same day or were frozen for later use. Spermatids were classified as round vs. elongated (6, 7, 8). Round spermatids were identified as slightly larger than red blood cells (approx. 8–10 μm) and with a visible acrosomal cap and large nucleus. Elongated spermatids were identified as cells slightly smaller than round spermatids with a visible sperm head attached to a cytoplasmic droplet. Spermatids were preisolated into microdrops of media before injection and were used only if no mature sperm were found. Preisolated cells were rinsed in 10% polyvinylpyrrolidone (PVP) before moving to the oocyte droplet for injection.

Surplus spermatids and tissue were cryopreserved for future ICSI attempts. The spermatid suspension was diluted with equal volume of sperm cryopreservation medium (sperm maintenance medium) and aliquoted into cryovials and suspended in liquid nitrogen vapour for 30 minutes before being submerged into liquid nitrogen for storage. For ICSI cycles using frozen-thawed spermatids, on the morning of oocyte retrieval, a vial of cryopreserved spermatids was thawed at room temperature for 20 minutes. Samples were subsequently prepared as described earlier.

### Ovarian stimulation, oocyte preparation, and ICSI

Ovarian stimulation and oocyte retrieval were undertaken using various individualized protocols. Data regarding stimulation protocols were not collected, nor was any attempt made to control for this variable. After oocyte retrieval, the oocytes were mechanically stripped of surrounding cumulus cells with the aid of hyaluronidase (MediCult, Jyllinge, Denmark), and ICSI was performed between 38 and 42 hours after human chorionic gonadotropin (hCG) or gonadotropin-releasing hormone (GnRH) agonist injection. The technique of ICSI was the same in both embryology laboratories in the study. Fertilization, defined as being 2 pronucleated (2-PN), was assessed 16–18 hours afterICSI, and the most developmentally advanced and morphologically normal embryos were subsequently transferred on either day 3 (cleaved to the 5- to 8-cell stage by 64–72 hours after injection) or day 5 (ideally cleaved to the blastocyst stage by 120 ± 10 hours after injection) after oocyte retrieval. Luteal support was either vaginal micronized progesterone suppositories or intramuscular progesterone injections from the day of transfer and continued until a negative β-hCG test or until the 8–10th week of pregnancy, where appropriate.

For clarity, blastulation rate is the number of blastocysts formed per fertilized oocyte; good-quality day 3 embryos are defined as 5–8 cells with minimum fragmentation; good-quality blastocysts are defined as 3AA or better (54), serum β-hCG levels are considered positive with levels >5 IU/L. Clinical pregnancy is defined as an ultrasound-confirmed intrauterine gestation, with a detectable fetal heart rate >100 beats per minute. Biochemical pregnancy is defined as a pregnancy in which a positive β-hCG level is identified, but a clinical pregnancy is not identified (most commonly where a delayed onset of a menstrual period occurred, before an ultrasound could be performed to confirm a clinical pregnancy). Miscarriage is defined as loss of a clinical pregnancy, before 20 weeks gestational age. Live birth is defined as birth of a child with a heart-beat and breathing at greater than 20 weeks gestational age.

### Reproductive outcomes

Outcome data were abstracted from patient chart records at MSH, and IVF-ICSI cycle-specific details from Mount Sinai Fertility and TRIO Fertility. The primary outcome was live birth, and secondary outcomes included successful fertilization, number of embryos available for transfer on day 3 or day 5, a successful embryo transfer, positive hCG, and clinical pregnancy. Elongated spermatids were characterized as either fresh or frozen-thawed. The ICSI cycles were linked to the mTESE procedure from which the utilized spermatids were obtained because some couples did multiple ICSI cycles from spermatids obtained from a single mTESE procedure. Additionally, any men who underwent more than one mTESE procedure during the study period were included separately for each mTESE procedure where spermatids were identified and utilized. Embryo transfers were either performed with fresh day 3 or day 5 embryos (if clinically appropriate), or with thawed embryos, either as the first or a subsequent embryo transfer. Data regarding frozen embryo transfer protocols were not collected; however, we did assess the impact of embryo transfer type on the overall results (see Results).

### Statistical analysis

Continuous variables were presented as means with standard deviations, and categorical variables were presented as counts with relative frequencies. Fisher’s exact test was used to assess differences between categorical variables. Significance was considered at *P*<.05. Binary outcomes were assessed using simple logistic regression both as an unadjusted model and adjusted for potential confounders including male age at the time of mTESE, female age at the time of ICSI, and number of oocytes inseminated. Odds ratios were reported with 95% confidence intervals. A fractional logistic regression was used for the percentage of fertilized oocytes. Sensitivity analyses were performed for fresh vs. frozen embryo transfers. Statistical analyses were performed using Stata version 18 (55).

## Results

### Male demographics and mTESE results

Male patient characteristics, including age, testicular biopsy results, hormone levels, medical issues potentially accounting for NOA and mTESE procedure results are reported in Table 1. The majority of men undergoing mTESE had some degree of testicular failure. Not all men underwent testicular biopsy before their mTESE procedures. All men included in the study underwent Y-chromosome microdeletion testing before the study. Microsurgical testicular sperm extraction was performed on one testicle in 35% of cases (right side in 62%, left side in 38%), and bilaterally in 65% of cases. The primary reason for extraction from both testes was inability to identify mature sperm in samples from the first testis sampled.

**Table 1:** Characteristics of patients undergoing mTESE and mTESE outcomes.

Characteristic	
Male age (y), mean (SD) (n = 256)	35.3 ± 5.5
Testicular biopsy result before mTESE (n = 256)	
No biopsy performed^a^	220 (85.9)
Biopsy performed^a^	36 (14.1)
Hypospermatogenesis^b^	2 (5.6)
Sertoli cell only^b^	16 (44.4)
Maturation arrest^b^	18 (50.0)
Hormone levels at time of mTESE, mean (SD) (n = 292)	
FSH (IU/L)(n = 281)	19.2 ± 12.3
LH (IU/L)(n = 280)	9.8 ± 6.7
Testosterone (nmol/L)(n = 280)	10.5 ± 5.7
mTESE procedures performed (per person), (n = 292)	
1	256
2	34
3	1
mTESE retrieval results, (n = 292)	
Mature sperm retrieved	97 (33.2)
Elongated spermatids retrieved	142 (48.6)
Only round spermatids retrieved	27 (9.2)
No sperm or spermatids retrieved	26 (8.9)
Medical issues in men with NOA using spermatids for ICSI, (n = 99)	
Idiopathic/undiagnosed	39 (39.4)
Varicocele ± embolization/repair	17 (17.2)
Cryptorchidism ± orchidopexy	11 (11.1)
Klinefelter syndrome	10 (10.1)
Chronic medical conditions	6 (6.1)
AZF mutations	4 (4.0)
Prior chemotherapy	3 (3.0)
Prior hernia repair	3 (3.0)
Pelvic trauma	2 (2.0)
Mumps orchitis	2 (2.0)
Chromosomal translocations	2 (2.0)

*Note:* All characteristics reported are for patients who subsequently underwent ICSI with elongated spermatids, except for the data on number of mTESE procedures performed and mTESE retrieval results, which include all patients in the overall cohort who had a mTESE procedure during the study period. Data presented as n (%) unless specified otherwise.

AZF = azoospermia factor; FSH = follicle-stimulating hormone; ICSI = intracytoplasmic sperm injection; LH = luteinizing hormone; mTESE = microsurgical testicular sperm extraction; SD = standard deviation.

a Percentage of total men undergoing mTESE.

b Percentage of men who had a testicular biopsy before mTESE.

### Female partner demographics

The average age for female partners who underwent ovarian stimulation, oocyte retrieval, and ICSI using elongated spermatids obtained from mTESE was 32.0 ± 3.9 years. A female infertility diagnosis was present in 21.6% of these women. The most common female factor infertility diagnoses were advanced age/diminished ovarian reserve (33%) and polycystic ovary syndrome (PCOS) (29%).

### ICSI cycle results

Results from ICSI cycles using elongated spermatids are shown in Table 2. One hundred thirty-eight ICSI cycles resulted from 99 mTESE procedures in which only elongated spermatids were found. Most ICSI cycles performed used frozen-thawed elongated spermatids (81.1%).

**Table 2:** ICSI outcomes using fresh vs. frozen-thawed elongated spermatids obtained from mTESE.

	Fresh spermatids	Frozen-thawed spermatids	
ICSI cycles performed (n = 138)	26 (18.8%)	112 (81.1%)	
Male age at mTESE (y), mean (SD)	35.5 ± 6.3	35.4 ± 5.2	*P* = .83
Female age at ICSI (y), mean (SD)	31.5 ± 3.9	32.2 ± 4.0	*P* = .24
No. of oocytes retrieved	304	1,183	
No. of oocytes injected	252 (82.9%)	965 (81.6%)	
No. of 2 PN embryos^a^	80 (31.7%)	350 (36.3%)	*P* = .29
No. of day 3 embryos obtained	41	200	*P* = .45
No. of day 3 embryo transfers	3	20	
Day 3 embryos transferred (mean)	7 (2.3)	31 (1.6)	
No. of blastocysts obtained	18	65	*P* = .08
No. of blastocyst transfers	8	40	
Blastocysts transferred (mean)	11 (1.4)	56 (1.4)	
Positive β-hCG (n)^b^	1 (3.8%)	8 (7.1%)	*P* = .79
Clinical pregnancy (n)^b^	1 (3.8%)	5 (4.5%)	*P* = .85
Miscarriages (n)^c^	0 (0)	2 (25%)^d^	*P* = .57
Live births (n)^b^	2 (7.7%)^e^	2 (1.8%)^d^	*P* = .36

*Note:*.

2PN = two pronuclei; β-hCG = beta human chorionic gonadotropin; ICSI = intracytoplasmic sperm injection; mTESE = microsurgical testicular sperm extraction; SD = standard deviation.

a % of oocytes injected.

b % per ICSI cycle start.

c % per positive hCG.

d Unknown miscarriage or live birth outcome x 1.

e Twin birth (each baby counted as 1 birth).

Progression to the blastocyst stage occurred in 33% of all ICSI cycles started. One embryo was cryopreserved on day 3 (1 ICSI cycle) and 19 embryos were cryopreserved at the blastocyst stage (13 ICSI cycles), representing cryopreservation in 10.1% of total ICSI cycle starts. All cycles in which embryos were cryopreserved occurred in cycles using frozen spermatids (data not shown).

A total of 71 embryo transfers (with 105 embryos transferred) occurred in the study cohort; of these, there were 61 fresh embryo transfers (86%), and 10 frozen-warmed embryo transfers (14%). Twenty-three ICSI cycles (34%) resulted in day 3 embryo transfer (all fresh), which was chosen because of low embryo number (usually 1 or 2) and wanting to avoid having no blastocysts to transfer. Forty-two ICSI cycles resulted in a total of 48 blastocyst transfers (66%), of which 77% were fresh. No embryo transfer occurred in 48.0% (n = 73) of cycle starts (data not shown).

Four live births occurred in the entire cohort (2.6% per ICSI cycle start, 4.0% per mTESE procedure). All live births resulted from embryo transfers of blastocysts from the first ICSI cycle performed, and all from fresh transfers. One set of twins occurred (each live-born child counted separately in live birth results), from fresh elongated spermatids with two day 5 blastocysts transferred.

Results of the regression analyses are presented for fresh vs. frozen elongated spermatids (Table 3). Freeze-thawing of elongated spermatids did not have any impact on reproductive outcomes compared with the use of fresh elongated spermatids. Adjusting for male age at the time of mTESE, female age at the time of ICSI, and number of oocytes obtained (as a surrogate for ovarian reserve) did not change any of the results. Adding fresh vs. frozen embryo transfer to the multivariable model did not significantly change the results.

**Table 3:** Regression analysis of fresh vs. frozen elongated spermatids on pregnancy outcomes.

	Unadjusted		Adjusted	
	Estimate	95% Confidence interval	Estimate	95% Confidence interval
Successful fertilization	OR = 1.18	0.87–1.60	OR = 1.14	0.84–1.56
No. of day 3 embryos	β = 0.19	−0.30–0.68	β = 0.29	−0.17–0.75
No. of blastocysts	β = −0.22	−0.46–0.02	β = −0.22	−0.47–0.3
Day of embryo transfer (Day 5 vs. Day 3)	OR = 0.88	0.21–3.76	OR = 1.52	0.70–3.30
Positive β-hCG level	OR = 1.36	0.15–12.21	OR = 1.69	0.17–16.67
Clinical pregnancy	OR = 0.80	0.08–7.70	OR = 0.61	0.05–7.00
Live birth	OR = 0.32	0.03–3.92	OR = 0.40	0.03–5.33

*Note:* Variables included in the regression analysis: male age at time of mTESE, female age at time of ICSI, number of oocytes inseminated (surrogate for ovarian reserve). β-hCG = beta human chorionic gonadotropin; ICSI = intracytoplasmic sperm injection; mTESE = microsurgical testicular sperm extraction; OR = odds ratio.

Comparison of results with patients who underwent mTESE and obtained mature sperm for use in ICSI within the clinical outcomes are included in Supplementary Data for reference (available online).

## Discussion

Because many studies (10, 12, 14, 19, 20, 21, 56, 57, 58), but not all (11, 13, 15), support the premise that cryopreservation of mature sperm does not affect reproductive outcomes after mTESE and ICSI, we hypothesized that there would be no difference in reproductive outcomes between fresh and frozen-thawed elongated spermatids. The current study confirmed this hypothesis for all outcomes analyzed. Controlling for confounders such as male and female age, ovarian reserve, and fresh or frozen embryo transfer did not change these results.

The findings of our study expand on the limited results of Sousa et al. (24), which found equivalent outcomes between fresh and frozen spermatids for fertilization of oocytes.

Similar to the report by Asanad et al. (23), a majority of the mTESE samples used for ICSI in this study used frozen-thawed elongated spermatids (81.1%). There are two important reasons for this. First, costly ovarian stimulation and oocyte retrieval could be avoided if the outcome of the mTESE procedure did not yield acceptable gametes for the couple (i.e., mature sperm/elongated spermatids). Second, access to the operating room required advanced booking, thus severely limiting the flexibility to time a fresh mTESE procedure the day of oocyte retrieval. In instances where the timing could be coordinated, fresh elongated spermatids were used. If not, then frozen-thawed spermatids were used. All mTESE procedures were performed by the same urologists, and there was no significant difference between their performance of the mTESE and the chance of using fresh or frozen-thawed spermatids. Similarly, both clinics had approximately 23% of ICSI cases performed using fresh spermatids, suggesting similar processes for timing ovarian stimulation cycles, and suggesting the usage of fresh spermatids was likely by chance.

Assessing contemporary success rates for the use of elongated spermatid use in ICSI is challenging because of the limited numbers reported in the literature and a lack of any recent reports, with essentially all studies reported between 1995 and 2003. Our 35.3% fertilization rate falls within the ranges previously reported (10%–71%) (39, 59). Our absolute live birth rate of 2.9% per ICSI cycle started (4.0% per mTESE performed) is low and difficult to compare with other studies because of the limited reported data but is likely similar to that reported in these prior studies.

The 33.2% mature sperm retrieval rate (SRR) from mTESE procedures in our study falls within the previously reported range of 18.4%–70.8% (60, 61, 62, 63). Because a diagnostic testicular biopsy has relatively poor predictive value for mTESE outcomes, our 25.3% biopsy rate in men undergoing mTESE is consistent with the trend of not offering biopsy before mTESE (64). Importantly, all men included in the study underwent Y-chromosome microdeletion testing before the study, and only a small number (4%) had an azoospermia factor (AZF) deletion (Table 1). Interestingly, only 18.1% of men with NOA in this cohort failed to obtain either mature sperm or elongated spermatids from their mTESE procedure. This represents approximately 82% of men undergoing mTESE in our cohort having a chance of obtaining gametes that could potentially be used for ICSI. This differs from prior reports where mature sperm and/or spermatid recovery rates were lower (39). This could be a helpful counseling tool for patients with regard to their expectations of having an attempt at a biological child from mTESE.

Because cryopreservation of mature sperm can have detrimental effects on sperm function, even if not necessarily compromising overall potential with ICSI, it is plausible that these same phenomena could also present in spermatids. First, sperm motility decreases after cryopreservation and subsequent thaw (65, 66). In addition, there is likely decreased fertilization potential with frozen-thawed sperm compared with fresh sperm in men with NOA who underwent mTESE and ICSI (13). However, when fresh and frozen immotile sperm were used, fertilization outcomes as well as pregnancy and live birth rates were similar (13). Because spermatids are nonmotile, this motility decrease phenomenon is likely less relevant. Sperm cryopreservation can also lead to potential oxidative stress-related damage to deoxyribonucleic acid (DNA) structure and epigenetic issues such as methylation disorders (65, 67, 68). There is conflicting data for the effects of these changes on direct reproductive outcomes and offspring health, but several studies support the notion that decreased fertility can result from increased oxidative stress in mature sperm (66, 69, 70, 71). What is unclear is whether spermatids have more, less or similar risk to mature sperm from the cryopreservation process, or whether poor reproductive outcomes using spermatids are related to freezing status/reactive oxygen species (ROS) exposure. Although beyond the scope of this study, some of the protective mechanisms present in mature sperm and the epididymis may be less relevant in spermatids; in fact, there may be more ROS protection in spermatids because defensive enzymes such as catalase, which are absent in mature sperm, are present in the peroxisomes in spermatids (72). Notwithstanding the above, because this was a retrospective study and the outcomes assessed were clinical in nature, there was no mechanism to assess whether any of these concepts were in fact occurring; however, but they raise some intriguing questions for future research.

Couples who did not proceed with ICSI after mTESE was performed did so almost exclusively because of the failure to retrieve mature sperm. However, other couples proceeded with ICSI using elongated spermatids. In most cases, this was because the use of donor sperm was unacceptable to them. Accordingly, a small subset of patients will be highly motivated to undergo mTESE and subsequent ICSI using spermatids because this approach represents their only acceptable option for family building. However, in many places, spermatid use has been abandoned as a treatment modality because of poor results and concerns about risks to the children born from the technique (73). Discussion of these specific risks is beyond the scope of this study, but several publications highlight some of these concerns on the basis of animal models and limited human data (74, 75, 76, 77, 78). Conversely, several other reports highlight the live birth outcomes using spermatids (both elongated and round), with reassuring results on testing of these children (31, 33, 39, 50, 52). The poor overall success rates and potential for abnormalities (albeit likely low) in the resulting children underscore the challenge with using spermatids for conception attempts and warrant a detailed discussion with patients regarding their use. Although not specifically applicable to this study population, for the above-mentioned reasons, the American Society of Reproductive Medicine has continued to deem the use of less mature round spermatids as experimental (79).

Strengths of this study include the following. First, the ability to compare both fresh and frozen-thawed spermatids in the same study adds to the body of data regarding the efficacy of using these various gamete preparations for ICSI in men with NOA who undergo mTESE. This has been poorly assessed in prior studies and now allows for more efficient planning of reproductive treatments with better expectation of outcomes. Second, the real-world applicability of how mTESE samples are utilized by different fertility clinics illustrates how success may be measured outside of highly specialized programs.

Several limitations also exist in this study. Most notably is that the sample sizes are still relatively small (although larger than those in most prior studies). This limits the statistical analysis of certain comparisons and prevents drawing firm conclusions in some cases. However, prior studies with limited patient numbers suffered from the same limitation. Second, because routine testicular biopsy was not performed before undergoing mTESE, and the mTESE samples were not assessed by testicular pathologists for a clinical/pathological diagnosis of the potential cause for NOA, classifying the men in this study into etiologic groups is not possible. This may limit comparisons with some other reported cohorts. At the two clinics involved in this study, clinical embryologists perform the sperm isolation and the ICSI procedures; these embryologists do not have extensive pathological expertise to make clinical diagnoses on the basis of the mTESE samples. We suspect this is true for most fertility clinics.

## Conclusion

In this cohort of couples with men who had NOA and underwent mTESE and ICSI with elongated spermatids at two different IVF clinics, overall reproductive outcomes were comparable with previously published reports, suggesting possible laboratory improvements because the originally published studies have not led to improved outcomes. There was no statistical difference in any reproductive outcomes between fresh and frozen elongated spermatids. This key finding allows clinics flexibility to use whichever approach is desired/feasible without concern for obvious adverse outcomes. However, because live birth rates using elongated spermatids were very poor at approximately 2.9% overall, we do not recommend use of spermatids with ICSI unless couples indicate they are unable/unwilling to use donor sperm, and only after careful discussion of the poor success rates and the potential risks to the pregnancy/child is undertaken.

## Declaration of Interests

J.E.E. has nothing to disclose. C.L. has nothing to disclose. J.S.M. has nothing to disclose. K.J. has nothing to disclose. E.M.G. has nothing to disclose.
