# Supplementary material for: Outcomes with intracytoplasmic sperm injection using fresh vs. frozen elongated spermatids from microsurgical testicular sperm extraction
Source: F S Rep. 2026 Aug 25;7(5):386–93. doi: 10.1016/j.xfre.2026.08.005 (PMC13681273; doi:10.1016/j.xfre.2026.08.005)
Supplement: Supplementary file 1 [file mmc1.docx]

Supplemental Material:

Table 1.	ICSI outcomes utilizing motile and immotile mature sperm obtained from mTESE

|  | Motile Sperm | | Immotile Sperm | |
| --- | --- | --- | --- | --- |
|  | Fresh | Frozen | Fresh | Frozen |
| No. of patients | 12 | 27 | 11 | 37 |
| No. of cycles | 12 | 33 | 11 | 44 |
| Male age at mTESE (y) | 35.6 | 39.5 | 41.3 | 37.5 |
| Female age at ICSI (y) | 32.6 | 33.6 | 35.3 | 34.1 |
| No. of oocytes retrieved | 143 | 379 | 142 | 496 |
| No. (%) of oocytes injected | 112 (78.3) | 337 (88.9) | 116 (81.7) | 421 (84.9) |
| No. (%) of 2 PN embryos* | 71 (63.4) | 197 (58.5) | 63 (54.3) | 202 (48.0) |
| No. (%) of blastocysts | 20 (28.2) | 91 (46.2) | 8 (12.7) | 31 (15.3) |
| No. of embryo transfers | 15 | 42 | 11 | 33 |
| No. (%) of blastocyst transfers | 12 (80.0) | 36 (85.7) | 7 (63.6) | 18 (54.5) |
| No. of embryos transferred (mean) | 22 (1.5) | 70 (1.7) | 23 (2.1) | 59 (1.8) |
| Positive βhCG [n, (%)]# | 3 (25.0) | 17 (51.5) | 1 (9.1) | 8 (18.2) |
| Clinical pregnancy [n, (%)]# | 3 (25.0) | 11 (33.3) | 1 (9.1) | 8 (18.2) |
| Miscarriages [n, (%)]& | 0 (0) | 1 (5.9)@ | 0 (0) | 3 (37.5) |
| Live births [n, (%)]# | 3 (25.0) | 9 (27.3)@,$ | 1 (9.1) | 6 (13.6)$ |

^*^ % of oocytes injected

^#^ % per ICSI cycle start

^&^ % per positive hCG

^@^ unknown miscarriage or live birth outcome x 1

^$^ Twin birth (each baby counted as 1 birth)

Table 2.	Unadjusted and adjusted regression analysis of ICSI outcomes utilizing fresh vs. frozen-thawed mature sperm.

|  | Fresh Sperm vs. Frozen Sperm | |
| --- | --- | --- |
|  | Unadjusted | Unadjusted |
| Having a blastocyst | OR 2.36 (1.33-4.23) | OR 0.94 (0.50-1.77) |
| Having an Embryo Transfer | OR 1.14 (0.62-2.09) | OR 1.08 (0.57-2.04) |
| Positive βhCG | OR 0.58 (0.24-1.41) | OR 0.54 (0.22-1.35) |
| Clinical Pregnancy | OR 0.72 (0.28-1.89) | OR 0.64 (0.24-1.72) |
| Live Birth | OR 0.98 (0.37-2.60) | OR 0.90 (0.33-2.44) |

Odds Ratio (OR)

* Adjusted for: Male age at time of mTESE, Female age at time of ICSI, # of oocytes inseminated

Table 3.	Adjusted regression analysis of ICSI outcomes utilizing mature sperm vs. elongated spermatids

|  | Adjusted | |
| --- | --- | --- |
|  | Estimate | 95% Confidence Interval |
| Successful fertilization | OR = 2.23 | 1.90-2.62 |
| Number of day 3 embryos | β = 2.22 | 1.88-2.57 |
| Number of blastocysts | β = 1.03 | 0.81-1.26 |
| Day of embryo transfer (Day 5 vs Day 3) | OR = 2.36 | 1.05-5.30 |
| Positive bhCG level | OR = 3.16 | 1.37-7.31 |
| Clinical Pregnancy | OR = 3.86 | 1.43-10.26 |
| Miscarriage | OR = 2.19 | 0.40-11.93 |
| Live Birth | OR = 12.29 | 1.48-101.67 |

Odds Ratio (OR)

* Adjusted for: Male age at time of mTESE, Female age at time of ICSI, # of oocytes inseminated
